# Thoughts upon Prescribing

**Published:** 1874-04

**Authors:** Frank A. Ramsey


Vol. IV.—APRIL, 1874.—No. 4.

### Original Communications.

### THOUGHTS UPON PRESCRIBING.

BY FRANK A. RAMSEY, M.D.

*Read before the Knox County Section of the East Tennessee Medical Society.*

The value of an agent employed for remedial purpose must be determined by the frequency of good results, taking into comparison the relative frequency and graveness of unpleasant effects, occasioned by its administration.

Heretofore, until at a comparatively recent period of time, the administration of medicinal agents was prompted wholly by the observations which have been made of effects following administration so that the material for medication has become huge in proportion to the aggregate of years that have been consumed in search for, and in the use of agents for remedial purpose. Very many of these have become as common with practitioners of medicine, as water is with mankind in general, and the administration of a me dicinal agent is ordered by the professional man, with as little consideration of its capacity, actual and relative, as water is used by individuals in households, and in communities. The consequence is, that very frequently there is no good result obtained by the use of a medicine; occasionally effects are more deleterious than the diseased action it was intended to stop; and, fortunately, more seldom, but yet with enough of frequency to be noticeable, death follows, when it would not have occurred if the agent had not been used under the circumstances.

In this view of the results from the use of the armament of the medical profession, it seems to be meet and right to call an association of practitioners, like this, to consider the subject of hasty prescriptions, and to the propriety of each one, as far as may be done, familiarizing himself with the possible effects, as well as with the ordinary effects, from the administration of agents, even of those with which he thinks himself to be most familiar.

The action of an agent upon the human economy, occasions effects, according to the conditions of the economy, and probably is largely influenced by relationships sustained by it for the moment.

Thus, an agent administered to a person in full health, would cause effects, perhaps, markedly different from the effects caused by the same agent, to the same person, in a state of disease; or, an agent known to impress the nervous system of the human economy, will cause certain pleasurable and beneficial effects when administered to a man whose bowels are soluble, but will cause certain unpleasant and injurious effects when administered to the same person at a time when his bowels are constipated, and have been so for a greater or lesser while; or, an agent may occasion pleasurable effects when administered to seventy-five of a hundred men, in full health, and very unpleasant and variable effects, or, perhaps, no perceptible effects on the other twenty-five. Each individual man has his own receptivity, and while the effects exhibited from the impression of the agent, on much the largest number of healthy individuals, must be regarded as the physiological action of the agent, the difference, and the variableness of effects, on the lesser number of healthy men, must be received as evidence of the quality of receptivity modifying the action of the agent, and practitioners must be on the watch to discover the character of that quality when observing the actions of medicines upon their patients, not forgetting, the meanwhile, a broad hint which has been given by a medical philosopher in published "notes and reflections," that it "is not too much to affirm that the judgment of the medical man upon a disorder, is often warped by the reflection of his own practice. He is apt to look at the symptoms through his own previous treatment, etc."

And remembering, too, an observation common to practitioners of medicine, which has been formulated by some one, thus: "On the physiological effects of an agent given to healthy men, it is impossible to predicate the identity, or even the analogy of the effects of the drug on men affected with morbid conditions."

As each man has his own receptivity, so each agent, of any class, has its own essential or individual energy, its own mode of producing effects, and this essential, this peculiar mode, ought, if possible, to be apprehended and appreciated, and be well considered, in making selections from a class to bring about a desired result, more closely studied when forming a prescription combining several agents. Holland says: "It may be alleged, and must be fully admitted, that combinations have often effects not resulting equally from any of their ingredients. This is true as respects many vegetable medicines of powerful actions, whether narcotic, purgative, diuretic or alterative. It is equally true as regards chemical medicines, where changes of combination may occasionally be calculated upon, favoring the effects desired; though much more frequently the facts by which their use is determined are merely empirical in kind. The observation of these facts is obviously one of the most important objects to the practitioner. But, it must be added, one of the most difficult also, for even in the simpler combinations we can rarely obtain that precise estimate of effects which is so essential to the success and certainty of practice—still less can we do so in those of a complicated kind. Each new ingredient added to a medicine, raises to a higher ratio the chances of error, and obscures the evidence by which such error may be detected and removed. And the application of other science to this subject (though never to be lost sight of) is here made so uncertain by being subjected to vital actions, that it must ever be admitted with great caution, and wholy in subordination to experience. Several compound medicines, of undoubted efficacy, contradict chemical laws even in the points where it might seem of greatest moment to maintain them. In adding to other difficulties the uncertainty of combinations, we wrap one doubt within another, and play false with every just principle of medical research." And it is pertinent to quote just here a remark made by Prof. Doughty, of Augusta, Ga.: "The clinical observer is not bound to explain the *modus operandi* of his remedies, however desirable that may be, but he is bound to employ them when their virtues are clearly established, without regard to his ability to explain them. The cure of disease is the *ultimathule* with him."

In no point is the acuteness of the practitioner's discrimination more heavily put to the test than in determining the dose of the agent proper for the individual instance of disease that gives him the occasion to prescribe. The books of authority mention minimum and maximum doses of medicinal agents, and give rules of proportions based on the age and sex of the patient. These can not be gainsaid as being relationships proper to enter into the considerations of the practitioner, but they are very far from being the only ones, and fully as far from being the most important relationships to be intimately balanced before prescribing. As the effects from agents differ as made from healthy and diseased tissues and economies, so too do the effects from different quantities of an agent as made from healthy or diseased tissues and economies. Anstie relates a case of nervous disease for which he directed less than three-fourths of a grain of an agent, in minute doses, distributed over seven days, at the end of that time, the patient exhibited the tonic effects of the agent; yet he refers to a previous communication from Mr. Ashburton Thompson, in which it was shown that the same agent will be borne by patients in doses from two to four times as as large as those which have been in use during the last few years. And in another paper on doses, he finds fault with the compilers of the English Pharmacœpia, giving instances where "the minimum dose of poisonous agents is far too large, and yet other instances in which the maximum dose is much too small for the production of desired results in many cases of disease. Thus it is that an authoritative book misleads practitioners of medicine, and institutes a timid and feeble style of administration that is very far from being a trifling evil."

These cursory generalizations serve to show the truth of the ancient maxim, that the whole art of medicine consists in observation, and sustains too the Hippocratic apotheme that observation is difficult.

It is very desirable for the practitioner to know just the tissue, and even the point of the tissue more positively impressed by the agents he employs to accomplish the end for which he is called to the bed-side of the sick; but it is essentially demanded of him, before administering an agent, to know the probable and the possible effects, and essentially demanded of him to be able to establish an adaptation of the probable effcts to the end he desires shall be attained through the action of the agent administered by him. It is manifest that this demand cannot be complied with by any one relying on his sole observation; and doubtless it was an appreciation of the impossibility in these premises that prompted Sir Astley Cooper to say that if he was deprived of the benefit of conference with his conferes, and of consulting the observations recorded by his predecessors, he would at once cease practice.

The remarks which have been made may be illustrated. There is no more efficient purgative than castor oil. It is an article of almost common consumption; is sold in retail stores of almost every designation; is not confined to the barrels of the druggist, or for dispensation by the apothecary; and is rarely absent from the pantry or medicine closet of the provident housewife. It is the ordinary substitute adopted by the practitioner of medicine, when the particular purgative agent his judgment suggests is too inconvenient to reach, or can not be readily obtained; and is ordinarily directed to follow after the lapse of a stated interval, the administration of some other agent; and has been frequently given to babes within the first hour after their births. It is true that all this is so, without there having been recorded any instance, perhaps, of any markedly bad immediate results. But within the observation of every practitioner, have not results transpired which he felt assured were unnecessary, and which he ascribed to an inconsiderate administration of castor oil? And, is not the very common use of the castor oil, without usually any very serious results, a cause for the non-recognition of a very bad result being ascribed to the action of castor oil, when in truth it is the most patent source for the unfortunate accident? Two instances readily occur to my mind as having presented under my own observation. A young lady, many years ago a resident of this city, had passed through a typhoid fever of several weeks' duration, and was said to have recovered. She had been out of bed for ten days or two weeks, and was visiting rooms adjacent to her own in the house—among them the dining-room. Her physician, of course, had quit regular visitations. One day, at dinner, she took a bit of remarkably fine apple-pie. It was affirmed to have been a very small bit, because of the apprehension of the family of its effects. The afternoon passed, and bed-time came, without any evidence of bad impression from the pie—but without any relief to the apprehensions of the family. These led them to administer a dose of castor oil on her retiring at night. Before daylight of the next morning, the great pain in her abdomen occasioned her physician to be recalled,—her bowels were inordinately purged, and always with griping, and by bed-time of that night, or twenty-four hours after the administration of the castor oil she was a corpse. There has never been a doubt in my opinion as to the source of death in this instance. The apple-pie had been taken fully eight, probably ten hours, without any evidence of bad result, and before the oil was taken; and, it was not more than four hours after she swallowed the oil before abdominal pain occurred. It can not be *post hoc propter hoc* conclusion, to deny pernicious results to the apple-pie, and ascribe them to the castor oil. Dr. Wm. Rodgers and myself had in charge a man in middle life, a farmer given to work, and who had enjoyed good health in every particular until his attack from typhoid fever. His sickness was prolonged through many weeks, and he was exceedingly weak, able only, I believe to move his eyes, perhaps his fingers, and to swallow. In this condition the fever left him, the diarrhœa of the attack had been stoped for two or three days, and he was taking nourishment with evident benefit. But officious neighbours visiting him learned that his bowels had not been moved for several days, urged the necessity, and succeeded in having a dose of castor oil administered, near sundown of a day. Before day-light his physicians were summoned because of abdominal pain, and bloody purgings, and before sundown of that day he seemed to be bleeding from every mucous surface of his body. By close attention on the part of his physicians, and the vigor of his own constitution, he did not die; but who will say the imminent peril in which he was placed, was not caused by the inconsiderate administration of castor oil? Every practitioner has witnessed the variableness of the operation of castor oil, sometimes purging kindly, sometimes, indeed often, occasioning intense and severe gripings, and not unfrequently, besides heavier actions than is desirable, inducing different degrees of straining, amounting, in some instances, to even severe tenesmus. A distinguished practitioner of the city of New York, in recommending aloes to be used for affections of the rectum, does not fail to ascribe very serious enhancement by the use of castor oil, to remove the constipation common in the troubles of that bowel. I am sure that I have seen several cases of rectal prolapsion in children, which I could not ascribe to any other cause than the persistent use of castor oil with them when they were but infants, and the subjeets of infantile constipation. Such observations can not but serve to deprecate the employment of the oil with new-born babes, as inflicting injury upon the, but beginning to be formed tissues, which perhaps may be overcome during growth, but which very probably, may be the basis of affections manifested by them further on in the progress of their struggles to continue to exist. The irritating quality of castor oil is ascribed to the hard husk of the bean that furnishes the oil, which is commingled with juice from the husk, subjected to the same pressure and at the same time with the pulp. Possibly different quantities of the oil have different proportions of the material obtained from the husk, and occasions a milder action from one dose, and severe, even violent action from another. The husk-juice may mot be diffused throughout the oil. This is only suggestive. As long ago as 1825, C. G. Holland, of England, suggested as a substitute for cod-liver oil, one drop of croton oil to an ounce of poppy oil, half an ounce for a dose, claiming for it more uniformity, and less violence in action.

Since the days of Paracelsus the different preparations of mercury have been, probably, more extensively employed for remedial end to a larger variety of symptoms, and dynamically recognized diseased conditions of the human economy than any other agents. Mercury, in any form, has met with opposition intended to displace it from the armament of the physician; but the intention has completely failed. It has received plaudits from many hundreds of close observers, astute reasoners, and skilled practitioners, so that its position as a "great good" is to-day upon so secure a foundation that the "tidal waves" which are, from time to time, set in motion for its destruction, but serve to wash away accumulation of error, and leave the character of the agent brighter, better understood, and more fully appreciated, even though the force against it is derived from scientific investigations, and promulgated as transactions of an influential medical organization. For many years mercury has been esteemed for its energy in correcting glandular inaction, and as causing elimination of bile. But a committee of Scotish scientists recently reported to an Edinburg society experiments made upon dogs, which prove, as far as they go, either negative results, or decreased secretery action of the liver under the influence of mercury. But the experiments were upon dogs, and upon dogs in good health; and, all agree can not be forced upon practitioners as producing results identical with the results that the agent would produce upon healthy men. The conclusions of the experimenters can not weaken the position of the agent in the confidence of practitioners whose observations, fortified by the observations of very many others, extending through a very long series of years, have demonstrated the efficiency of the agent as opposed to diseased action in the human economy. Doughty, of Georgia, has correctly criticised the experiments and conclusions of the Edinburg committee; his article is commended to the attention of every practitioner of medicine.

But, after all, is this agent always given with due consideration by practitioners? Many years ago I was acquainted with a physician of reputation and doing extensive practice, who taught his pupils, "when at a loss what to do, or what to administer, order small doses of calomel and ipecac." A teaching that makes a plaything of a more than an edged tool; inculcates looseness of morals in as much as it lessens the graveness of responsibility appertaining to the functions of the physician, and induces him to think but little, if at all, when seemingly investigating a case of disease. How many practitioners are there who have never heard of the enunciated teaching, but who have practically adopted it, and, relying upon the protean qualities of mercury—sure of some effect from its presence in the human economy, administer it without a moments reflection, and when asked for what purpose it is given, ensconce themselves behind the vague reply "as an alterative."

Let each practitioner tell himself how often he has known others to order, how often he himself has prescribed, a form of mercury under circumstances for which there was no precedent, and as an experiment no reason, that could be pointed to or related. There is, perhaps, no substance which, in chemical combinations, so well illustrates the possession of intrinsically different qualities, or essentially differing modes of producing results. Itself, said to be incapabel of making any impression upon the tissues of the human economy, has developed the most varied qualities, and various degrees of force, by union with other entities, with which it has affinities, forming a variety of agents, each having its own intrinsic capacity, and producing results after its own essential mode of operating. And, in common with many other agents, it, in its chemical combinations, occasions different results according to the dose administered; and the effects of dosage are modified by the frequency of repetition; but yet in any dose preserving the capacity of impressing the glandular system, the liver, being the largest gland, exhibiting results from impression more noticeable, perhaps, than from any other part of the economy. It can not be otherwise than that a dose of a mercurial preparation when administered unnecessarily will make an impression followed by results long after the dosing has been forgotten, probably ulterior results after the agent has been fully eleminated from the economy—and certainly ulterior effects proportioned to the frequency and persistence of the repetition of the agent. Three instances of profound nervous trouble, doubtless involving material lesion, have occured under my observation. Many years ago I was called to visit Dr. Milton Tate, at his residence in Clinton, Anderson county, Tenn. At the time he was about forty-seven years of age, was a large muscular man who had lived much out of doors, practicing medicine and hunting. Had never been confined to bed by sickness, and had never drank a pint of whiskey, wine or brandy during his life. Had been much troubled for very many years with a varicose ulcer of the leg. But during the twenty years of his more active and arduous life as a practitioner, he affirmed that not one week had passed without his taking five grains, and frequently more, of either blue pill or calomel, and sometimes the two agents together. At the time of my call he was in bed with migratory paralysis. He said his habits of life had been so uniform and of such a character, that he could not ascribe the peculiarity of his attack to anything but the persistent use of mercury. He had always feared biliary inaction, but never mercurial paralysis, but that now he felt that his confidence had been misplaced, and the agent to which he had appealed to prevent him injury, was proving itself the source and cause of his destruction. He died suddenly, evidently with paralysis of the heart. I detailed the history of the case to my then neighbor and friend, Dr. Wm. J. Baker, whose great apprehension in life was liver inaction, and who, to my own knowledge was accustomed to taking mercury in some form, with a frequency that induced me very often to remonstrate with him, and I used this instance of Dr. Tate, with which to add force to my opinion, predicting Dr. B's death by paralysis, if he did not refrain from the almost constant use of mercury. He died paralized, occasioned largely, as I believe, by the previous use of mercury. The facts are given and others may entertain different opinion. An educated man, a successful lawyer and a politician with national reputation, had lived soberly all his life. Had never been sick, and never indulged in even so much as a glass of wine. However, on each and every occasion that he had to make an important speech, or to demean himself with quickness and correctness, and with impressiveness, he was accustomed to take twenty grains of calomel, so timed that his glandular system would be intensely active during the time he was engaged in speaking. And whilst occupying the position of Speaker of the U. S. representative branch of Congress, it is said, he took smaller doses of calomel regularly. His political life terminated, and he begun duty as a Circuit Judge. But hardly two courts passed when he scattered his brains, by shooting himself with a double-loaded pistol. The habitual use of calomel was the only error of habit in his life, and without attempting a rational—connecting it as prime cause in the ultimate result, I can not think that any other source for the end can be correctly determined.

When I begun practice, fresh from school, I was impressed with my ability to wield this huge instrument of warfare, to appropriately direct this Sampson of the materia medica, with an effectiveness which I do not feel to-day regarding any means employed by the physician. I was familiar with the almost uniform prescription, in small doses, of calomel and ipecac, made by practitioners of medicine everywhere, in the diarrhœa of nurslings, of dentition, commonly called, without, perhaps, differentation, cholera infantum, as I was familiar with the almost uniform failure, on the part of the prescribers, to discover any radical good following its administration; and, I was too fully crammed with the doctrine of one of my public teachers, starting with biliary involvement as the essentiality of the disease—without, for a moment, recognizing my ignorance of the essential nature of the biliary involvement, but sure that twenty grains of calomel would prove a sword both strong and keen to destroy any gordian knot, and bring into manifest view the curative influence of my dose of the agent, in its energetically promoting the emunctory function of the liver, displayed in the large, thickened yellow, or brownish-yellow matters discharged from the bowels, with marked decrease in the frequency of bowel-movement.

I don't know that I have ever had an instance of observation to make me regret having begun practice on this particular line in this particular encounter; certainly none to place in comparison with the number of observed instances of injury indubitably resulting from the protracted administration of calomel in doses of $\frac{1}{8}$ a $\frac{1}{4}$, and a $\frac{1}{2}$ grain, alone or combined—but I am free to say that I am now as much astonished at my own temerity as at the successful results following its exercise.

I remember visiting at the Paper Mill in this county, a child twenty-two months old. My first visit was made during the month of October, when I was told that the child had first sickened during the previous April, and had been treated with chalk mixture, rheubarb and chalk and camphor, milk in which blackberry and dewberry roots had been boiled, and with long continued small doses of calomel and ipecac. It was lying in its cradle with legs drawn up, feet crossed, retracted abdomen, cold extremeties, half open, deeply sunken eyes, open mouth, thickly coated, red tiped tongue, respiration and pulse so rapid that I did not presume to count them, and, with all, vomiting and purging every time water or anything was put into its mouth. I promptly and freely scored the gums. Accurately weighed twenty grains of calomel, and laid it upon the child's tongue and washed it down by rapidly putting a teaspoonful after teaspoonful of water into the patient's mouth. In the mean while they had prepared a gallon of a decoction of red and and yellow Peruvian barks, to which was added a quart of proof corn whiskey. In this, at a temperature of 90° F., the child was bathed for a very short while. The barks, from which the decoction had been made, were put into the form of poultice, with meal and hot whiskey, and placed, well secured, over the child's abdomen. Diet, as wine whey, milk with camphorated lime-water, thin arrow-root with wine, teaspoonful doses of one, or another, at intervals not shorter than half an hour, nor longer than one hour, with water during the intervals if lips or tongue seemed dry, was ordered. The child never vomited after the gums had been freely lanced, and the calomel was weighed and administered after the operation, so that several minutes must have elapsed between surgery and dosing in the case. Recovery was rapid and complete. I have been somewhat prolix in giving the treatment, because I desire you to appreciate a doubt now, not then, on my mind as to the probable effect of the calomel. Did it do any good? Was it necessary? I regarded it as the potent agent, without which the patient could not have lived; but, in fact, were not what I regarded as mere adjuvants, mere auxiliaries, the powerful sources from which resulted the stay of diseased process, and of death?

As pertinent, since the introduction of such questions, permit here the details of another case. During the Summer of 1870, the proprietor of a job printing office, in Memphis, asked me to see his son, aged twenty months, who had been sick six weeks. He had been the whole time taking quinine and calomel alone, and in various combinations, but always in small doses.—Finally, symptoms of brain trouble presented, and the attending physician, one properly enjoying large confidence, and doing extensive practice in Memphis, directed a blister to be applied to the nape of the neck. The mother objected; a change of medical advisers resulted. I found the child had seemingly fallen off but little, yet with soft, flabby tissues; hot surface; very rapid breathing; each expiration slight, apparently suppressed; groans; pulse 140; swollen abdomen, and diarrhœa, with frequent vomitings; red, parched lips; dry tongue with brownish fur; head thrown back, whether the child was placed on its side or back; eyes half-closed, and very positive listlessness; "always worse during the early morning, but for ten days has been growing worse until now he's just as bad as you find him, as bad as he can be to live." I have quoted from the mother. I immediately and freely lanced the boy's gums, without his giving evidence during the operation of any unusual impression; but in less than ten minutes afterward he opened his eyes, asked in his own mode for water, raised himself from his mother's arms, and drank freely, then amused himself, much to his mother's delight, investigating the two strangers in the room. I directed all medicines, except the quinine, to be discontinued, ordered an alkaline poultice to the abdomen, and allowed ice or water at the child's pleasure, suggesting that it would be proper to offer it to him frequently, but never to be forced upon him. I received reports from him for two or three days, but had no occasion to make a second call. During the Summer of 1872 I saw the same boy, heartily enjoying himself, in fine health. Doubtless, similar differences of treatment in the same class of cases will readily recur to the memory of each practitioner of medicine, who has ever permitted himself to doubt the absolute propriety, or the necessity of any prescription he may have made; and that, too, without causing him to waver a moment in his confidence in the beneficence of the art which he practices, and the curative energy, when properly applied, of the agents that he employs; but, the rather, causing him to gird up his loins, and with renewed vigor to go on the prosecution of the work of his life, and by pure, close, intense observation, attempt to master the relationships of things, that he may the better conquer the ills of mankind, which he is appointed to encounter—causing him not to indulge in apathetic reliance on an asserted fact so long as a doubt can be entertained of its correctness, but questioning all things, proving all things, and holding fast only to that which, in the fullness of man's fallible judgment, he is satisfied is molifying or curative, from "the nature of the disease, or from the character of the agent as a specific, or from pure empiricism."

It is, I believe, the concurrent observations of practitioners of medicine that a scruple of calomel will occasion much less perturbation than a half or a fourth the quantity. The lesser quantity will occasion several acrid and griping stools, perhaps requiring the administration of an opiate to destroy the unnecessary and unpleasant effects; while the larger quantity will occasion but one or two large, discolored, fœted, consistent discharges, and ordinarily without effort on the part of the patient, and without the occurrence of pain. Hence, very many physicians regard the smaller doses of calomel as irritants, and the larger doses as just the opposite—as allaying pains, as sedative to the mucous membrane of the first passages. Such was the view of that prince amongst practitioners, of his day, Cartwright, of Mississippi, who ascribed bad effects only to small doses of calomel, and who successfully treated mercurial salivation, even the very worst cases, with twenty-grain doses of the "mild muriate of mercury."

But the condition of a patient under the action of one and another disease, or on a system on which disease has peculiarly acted, will modify the impressions of calomel, so that even small doses are not irritants, but are harbingers of joy, when in their absence the darkness of despair hovers in the sick-room because of the incompleteness of the physician's means to eradicate disease, or thwart its disastrous effects. How often those of us who have had much contest with typhoid fever, have been made to rejoice in a joy that knew no abatement, by administering, after the suggestion of Graves, of Dublin, a simple grain of calomel, and, perhaps, repeating it once or twice after intervals of twenty-four hours? The clean, red glazed tongue had disappeared, and it lay broad and furred in its bed, whilst the whole man of the patient delayed giving evidence of recuperation; no longer did there exist any necessity for administering heat-molifying resources; no necessity for presenting the cooling iced draughts, but proper nourishment and appropriate stimulants failed to induce any sign of advance in improvement, when, as by the magician's presto, a single grain of calomel opened up afresh the waning hopes, and perhaps, one and another grain, after long intervals of time, secured a rapid progress, until health was attained.

There are many other agencies ordinarily employed by the physician, which could be made to illustrate the single idea intended to be conveyed and impressed by this paper, of the absolute necessity of profound consideration on the part of the practitioner whenever he orders the administration of any medicine. But I will close by quoting Troussaise's remarks on opium, that have been called to my attention since I began the preparation, in form, of these thoughts upon prescribing. Trousseau says; "To derive from opium all the benefit it is capable of imparting, it requires to be given with the greatest circumspection. It is impossible for me to tell you the exact dose in which it ought to be administered. In each particular case the physician must decide this question considering the tolerance of the individual for oipum. There exists great diversity in this respect, not merely in the difference in tolerance in individuals, but also in the difference between the degree of tolerance which the same person has at different times, according to the varying circumstances in which he may happen to be placed. Some persons can bear enormous quantities of opium; and I mentioned remarkable examples of this peculiarity when lecturing upon epileptiform neuralgia. Others are affected by a single drop of laudanum; this statement is applicable to adults; but young children are sometimes narcotised by even one-fourth of that quantity. Nothing is so difficult as to judiciously manage opium. On this fact I cannot lay too much stress; for no remedy is dispensed so improperly, so prodigally, and with so little inquiry into the idiosyncracy of the patient. Note well, gentlemen, that this observation has a general bearing, and does not only apply to what is done in the treatment of a particular disease. During my clinical lectures I shall have frequent opportunities of raising my voice against this abuse."

